# Risk-reducing surgery in BRCA 1/2 mutation carries: a point of view

**DOI:** 10.18632/oncotarget.6348

**Published:** 2015-11-18

**Authors:** Francesca De Felice, Claudia Marchetti

**Affiliations:** Department of Radiotherapy, Policlinico Umberto I “Sapienza” University of Rome, Rome, Italy

**Keywords:** risk-reducing surgery, BRCA, mutation

BRCA1/2 mutation identifies healthy women who are at 5 to 20 fold increased risk for future development of ovarian cancer (OC) and breast cancer (BC) [1]. In general, BRCA 1 mutation carriers have an elevated lifetime risk of BC (65-85 %) and a slightly lower associated lifetime risk of OC (20-50%); whereas in BRCA 2 mutation it is reported an OC incidence of 10-15 % and a similar BC risk as BRCA 1 [1].

We would like to provide a reliable evaluation of the effect of risk-reducing surgery, both salpingo-oophorectomy (RRSO) and bilateral mastectomy (BRRM), to reduce long-term onset of ovarian cancer OC and/or BC in BRCA 1 / 2 mutation carriers. Considering the recent two meta-analysis [2-3], a strong efficacy of RRSO and BMMR strategies in OC and BC risk-reduction has been respectively confirmed (80% and 94%). Adding new data did not change the magnitude of the known impact of RRSO on OC risk reduction [4], as well as BMMR on BC [5], whereas it was demonstrated the benefit of BMMR also for those patients who received both surgical procedures. Consequently these results should be useful to determine standard risk-reduction management in this setting of patients, as well as generating new hypotheses to be proposed in clinical practice.

Three separate questions can be raised to address the variety of approach: firstly, the choice of both risk-reducing surgies in patients with BRCA 1 / 2 mutation, secondly the optimal timing in performing surgical strategies and lastly the cost-effectiveness of prophylactic surgery.

Regarding the first question, because of RRSO decreases the risk of OC by 80% and also reduces the risk of BC up to 50%, nowadays, it is recommended by the age of 40 years after the completion of childbearing [6]. But the residual BC risk in post-RRSO women remains still higher (up to 40%) than that in general population [3]. This residual risk, in addition to the absolute higher lifetime risk of BC than OC (56-84% versus 10-46%) and the evidence that BRRM nearly eliminates the risk of BC, should be considered sufficient to justify BRRM. In addition, we have no randomized data that compared survival benefit between BRRM and screening regimens. Importantly, there was a Monte Carlo simulation model suggesting prophylactic surgeries as cancer risk-reducing options [7]. Breast screening plus RRSO appeared to yield lower survival probability (6% and 3% in BRCA1 and BRCA2, respectively) with an increase in incidence (46% and 36% in BRCA1 and BRCA2, respectively).

The question of the optimal timing of BRRM and RRSO is still controversial.

Women with BRCA 1/2 mutations are more likely to develop BC at a younger age than OC [8]. The best solution to maximize survival and minimize cancer risk should be to perform both risk-reduction surgeries at 25 years old [7]. Otherwise the important possible long term sequelae related to hormonal replacement treatment (HRT) reinforce the evidence-based guidelines to performed RRSO by the age of 40 years, or at least once childbearing is complete. In contrast, BRRM should be performed at age 25 years, due to its capability to nearly eliminate the risk of BC. The fact that there is an excess of esthetic plastic breast surgery strongly suggests that this surgery is safe, without deleterious effect on life.

Finally the cost-effective consideration. Clinical practice guidelines are usually based on cost-effectiveness. As a result, BRRM plus RRSO approach maybe inherent to the maximization of survival probability is difficult to reconcile with the necessity to demonstrate a decisive economic advantage over the screening and surveillance program. It is true that a significant number of BRCA 1 / 2 mutation carriers will never develop cancer. But, on the other hand, the probability that BRCA 1 / 2 mutation carriers who choose regular intensive surveillance will develop a BC requiring multidisciplinary treatment approach, as well as prophylactic controlateral mastectomy, should be evaluated [8].

Probably the relevant question is whether BRRM can reduce anxiety in this setting of patients. Psychological stress and women social background, including family cancer history and personal life experience, could have a substantial influence on patient choice. Precision in reporting risk-benefit issues may contribute to facilitate patient's decision preference.

In conclusion, the management in BRCA 1 / 2 mutation carriers should be considered paramount and prevention strategies will be a dynamic area of research programs. The decision about prophylactic interventions remains highly personal, driven by women priorities. However the weight of the evidence suggests that the performance of both risk reduction surgeries could be a valid option to discuss with full knowledge of the facts.
